# Complete Genome Sequence of *Flavobacterium psychrophilum* Strain CSF259-93, Used To Select Rainbow Trout for Increased Genetic Resistance against Bacterial Cold Water Disease

**DOI:** 10.1128/genomeA.00889-14

**Published:** 2014-09-18

**Authors:** Gregory D. Wiens, Scott E. LaPatra, Timothy J. Welch, Caird Rexroad, Douglas R. Call, Kenneth D. Cain, Benjamin R. LaFrentz, Benjamin Vaisvil, Daniel P. Schmitt, Vinayak Kapatral

**Affiliations:** a National Center for Cool and Cold Water Aquaculture, Agricultural Research Service, United States Department of Agriculture, Kearneysville, West Virginia, USA; b Research Division, Clear Springs Foods, Inc., Buhl, Idaho, USA; c Paul G. Allen School for Global Animal Health, Washington State University, Pullman, Washington, USA; d Department of Fish and Wildlife Sciences and the Aquaculture Research Institute, University of Idaho, Moscow, Idaho, USA; e Aquatic Animal Health Research Unit, Agricultural Research Service, United States Department of Agriculture, Auburn, Alabama, USA; f Igenbio, Inc., Chicago, Illinois, USA

## Abstract

The genome sequence of *Flavobacterium psychrophilum* strain CSF259-93, isolated from rainbow trout (*Oncorhynchus mykiss*), consists of a single circular genome of 2,900,735 bp and 2,701 predicted open reading frames (ORFs). Strain CSF259-93 has been used to select a line of rainbow trout with increased genetic resistance against bacterial cold water disease.

## GENOME ANNOUNCEMENT

Infectious disease causes significant losses in aquaculture, and bacterial cold water disease (BCWD) is a common cause of rainbow trout loss (1, 2). Disease resistance can be improved through selective breeding (3, 4), and recent farm trials of a selectively bred line (ARS-Fp-R) demonstrated significantly higher survival at locations where BCWD is endemic (5, 6). *Flavobacterium psychrophilum* is the causative agent of BCWD and a reference genome sequence is available (7). Herein we report the complete genome sequence of *F. psychrophilum* strain CSF259-93 utilized in the National Center for Cool and Cold Water Aquaculture (NCCCWA) rainbow trout selective breeding program. The CSF259-93 strain has been used to derive a live attenuated vaccine (8, 9) and has been characterized biochemically and immunologically (10–16). The strain belongs to multilocus sequence type 10 (17).

A single colony of strain CSF259-93 was subcultured and grown in tryptone yeast extract salt (TYES) broth, and a large stock was stored at –80°C for challenge and DNA isolation (5). Isolated DNA was sheared and a random library prepared and sequenced using the Sanger di-deoxy method to 8× coverage. Sequences were assembled using larger contigs with *de novo* assembly with the Phred-Phrap-Consed package. The contig scaffolds were closed using a 40-kb fosmid library and end sequencing by use of the Sanger di-deoxy method followed by gap closure using PCR. The genome was closed to a single chromosome and assembly validated by optical mapping using NcoI. The genome of strain CSF259-93 is 2,900,735 bp, with an average G+C content of 32% and 2701 open reading frames (ORFs) including 49 tRNAs species and 6 rRNA operons. The genome was analyzed with the ERGO genome application platform (18), using previously described annotation methods (19), and 63% of the ORFs were assigned functions.

Comparative sequence analysis identified 2,481,839 bp common between CSF259-93 and strain JIP02/86 (7), with 1,471 single nucleotide polymorphisms (SNPs) present within protein-coding ORFs (617 nonsynonymous SNPs), 4 SNPs within RNA ORFs, and 119 SNPs in noncoding DNA. Five notable regions of difference were identified between strains. There is an expansion of 19 tandem leucine-rich repeat genes in CSF259-93 (nucleotides [nt] 215,399 to 235,946) compared to 15 tandem genes in the JIP02/86 genome. The number of repeats within each gene varies from 3 to 11, with each repeat encoding ~23 amino acids. This locus exhibits rearrangement in gene synteny and contains an overabundance of nonsynonymous SNPs (*n* = 64 out of 99 putative SNPs). There is an ~1.9-Mb chromosomal inversion between the putative adhesins, FPSM_00355 and FPSM_02084. There is a large genomic island of ~146,000 kb, a segment (597,708 to 743,794) present in the CSF259-93 genome containing 26 transposase ORFs, type II and III restriction modification systems, two putative tetracycline resistance genes (FPSM_00635 and FPSM_00640) (20, 21), and an integrase (FPSM_00578) absent from the JIP02/86 genome. There is a substitution of several lipopolysaccharide (LPS) biosynthesis genes (FPSM_02190 through FPSM_02194 and FPSM_02202). Finally, strain CSF259-93 lacks an integrated prophage that is found in the JIP02/86 genome (22).

The availability of complete and draft genome sequences combined with laboratory challenge data will facilitate definition of host specificity and mechanisms of bacterial cold water disease resistance.

### Nucleotide sequence accession number.

The genome sequence for *Flavobacterium psychrophilum* strain CSF259-93 has been deposited in GenBank under the accession number CP007627.
